# Supplementary material for: Paracrine FGFs target skeletal muscle to exert potent anti-hyperglycemic effects
Source: Nat Commun. 2021 Dec 14;12:7256. doi: 10.1038/s41467-021-27584-y (PMC8671394; doi:10.1038/s41467-021-27584-y)
Supplement: Supplementary file 1 — Supplementary Information [file 41467_2021_27584_MOESM1_ESM.pdf]

Supplementary Materials

Paracrine FGFs target skeletal muscle to exert potent anti-hyperglycemic effects

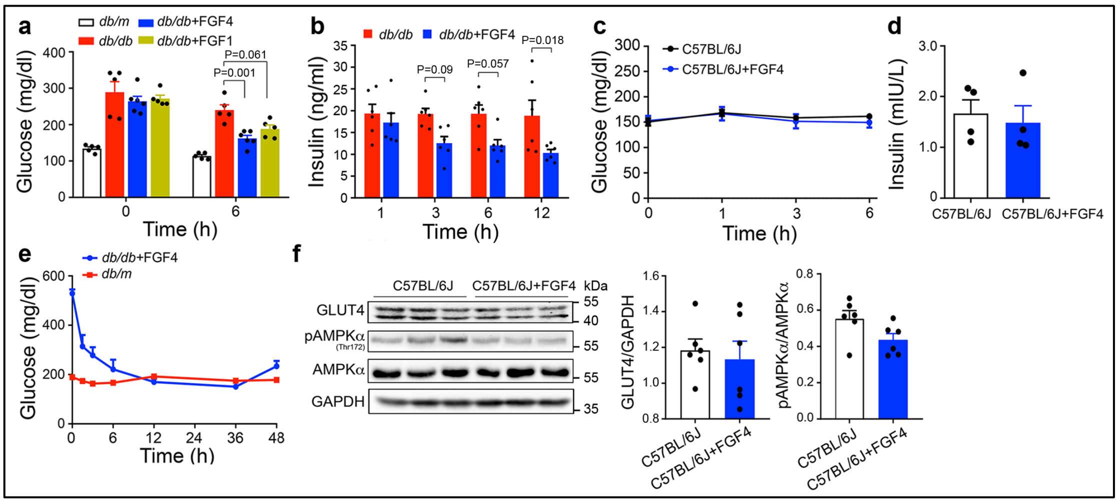

Supplementary Fig. 1. rFGF4 exerts FGF1-like effects on blood glucose and insulin levels in *db/db* and in wild type mice.

(a) Blood glucose levels in fasted *db/db* mice before and 6 hrs after a single i.p. injection of rFGF4 (n=6) or rFGF1 (n=5) (each at 1.0 mg/kg body weight) or PBS buffer as control (n=5). PBS-injected *db/m* mice served as an additional control (n=5). **(b)** Insulin levels measured at various times in *db/db* mice after acute i.p. injection of rFGF4 or a buffer control (n=6). **(c)** Changes in blood glucose concentration following a single i.p. injection of rFGF4 in wild type (i.e., C57BL/6J) mice (1.0 mg/kg body weight) (n=4). **(d)** Insulin levels in wild type mice 6 hrs after a single i.p. injection of rFGF4 (1.0 mg/kg body weight) or PBS buffer as a control (n=4). **(e)** Changes in blood glucose concentration following a single i.p. injection of rFGF4 in *db/db* mice (3.0 mg/kg body weight) (n=6). **(f)** GLUT4 expression and AMPKα phosphorylation levels in lower limb muscle (LLM) of wild type mice before and 6 hrs after i.p. injection of buffer (control) or rFGF4 (1.0 mg/kg body weight) as determined by western blotting (left panel) and quantitated using ImageJ software (right panel) (n=6). Data are presented as mean +/- SEM. Statistical comparisons in (a, b) are two-way ANOVA tests with Tukey's multiple comparisons test (a) or Šídák's multiple comparisons test

(b). Source data are provided as a Source Data file.

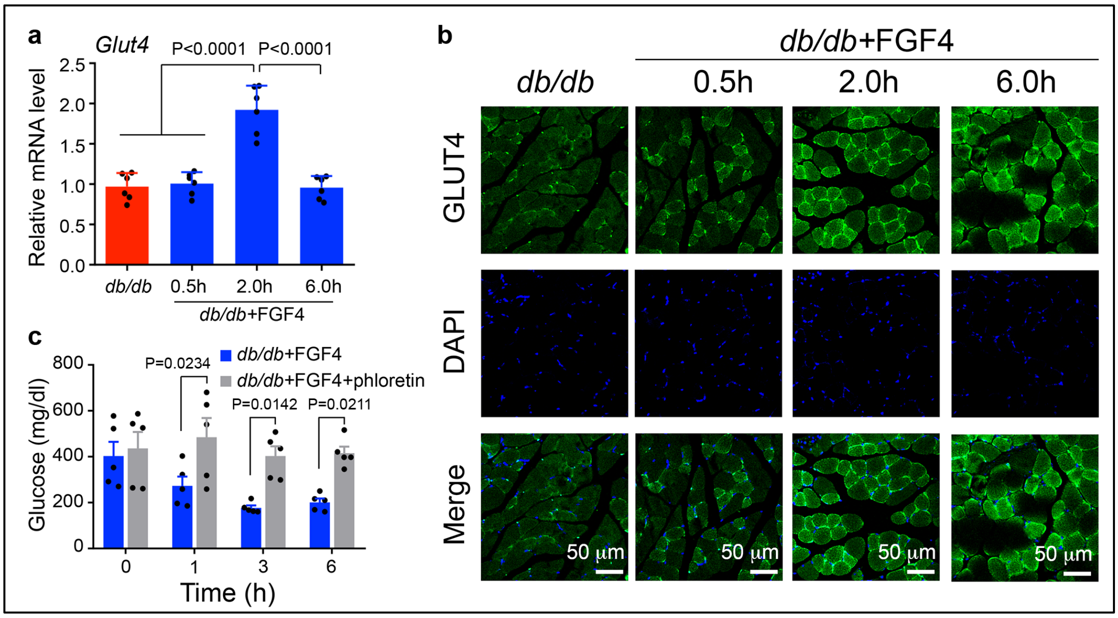

Supplementary Fig.2. Short-term rFGF4 administration increases transcription and protein translation of GLUT4 as well as its translocation to skeletal muscle cell membranes.

(a) mRNA level of GLUT4 in LLM of *db/db* mice at various times after a single i.p. injection of rFGF4 (1.0 mg/kg body weight) or PBS buffer (control) as determined by real-time PCR analysis (n=6). **(b)** Expression and translocation of GLUT4 in LLM of *db/db* mice at various times after i.p. injection of rFGF4 (1.0 mg/kg body weight) measured by immunofluorescence staining with an antibody to GLUT4. PBS buffer served as controls. Data are representative of 4 mice from each group. Scale bars, 50 μm. **(c)** Blood glucose levels after i.p. injection of rFGF4 (1.0 mg/kg body weight) into *db/db* mice pretreated for 1 hr with phloretin (200 mg/kg body weight) (n=5). Data are presented as mean +/- SEM. Statistical comparison in (a) is one-way ANOVA test with Tukey's multiple comparisons test. Statistical comparison in (c) is two-way ANOVA test

with Šídák's multiple comparisons test. Source data are provided as a Source Data file.

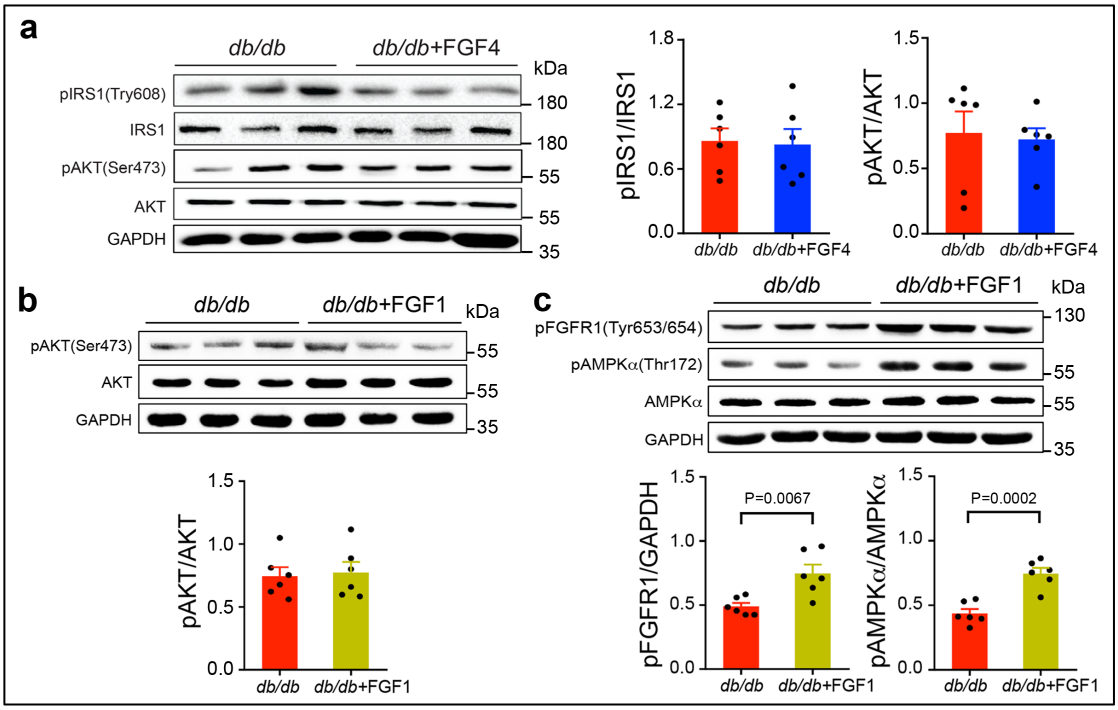

Supplementary Fig. 3. Both rFGF1 and rFGF4 fail to activate the insulin signaling pathway in skeletal muscle.

(a) Phosphorylation levels of IRS-1 and AKT in LLM of *db/db* mice 6 hrs after a single i.p. injection of rFGF4 (1.0 mg/kg body weight) as measured by western blotting (left panel) and quantitated using ImageJ software (right panels) (n=6). **(b,c)** Phosphorylation levels of AKT **(b)** and FGFR1, AMPKα **(c)** in LLM of *db/db* mice 6 hrs after a single i.p. injection of rFGF1 (1.0 mg/kg body weight) as measured by western blotting (upper panels) and quantitation using ImageJ software (lower panels). GAPDH was used as a loading control (n=6). Data are presented as mean +/- SEM. Statistical comparison in (c) is unpaired two-tailed test. Source data are provided as a Source Data file.

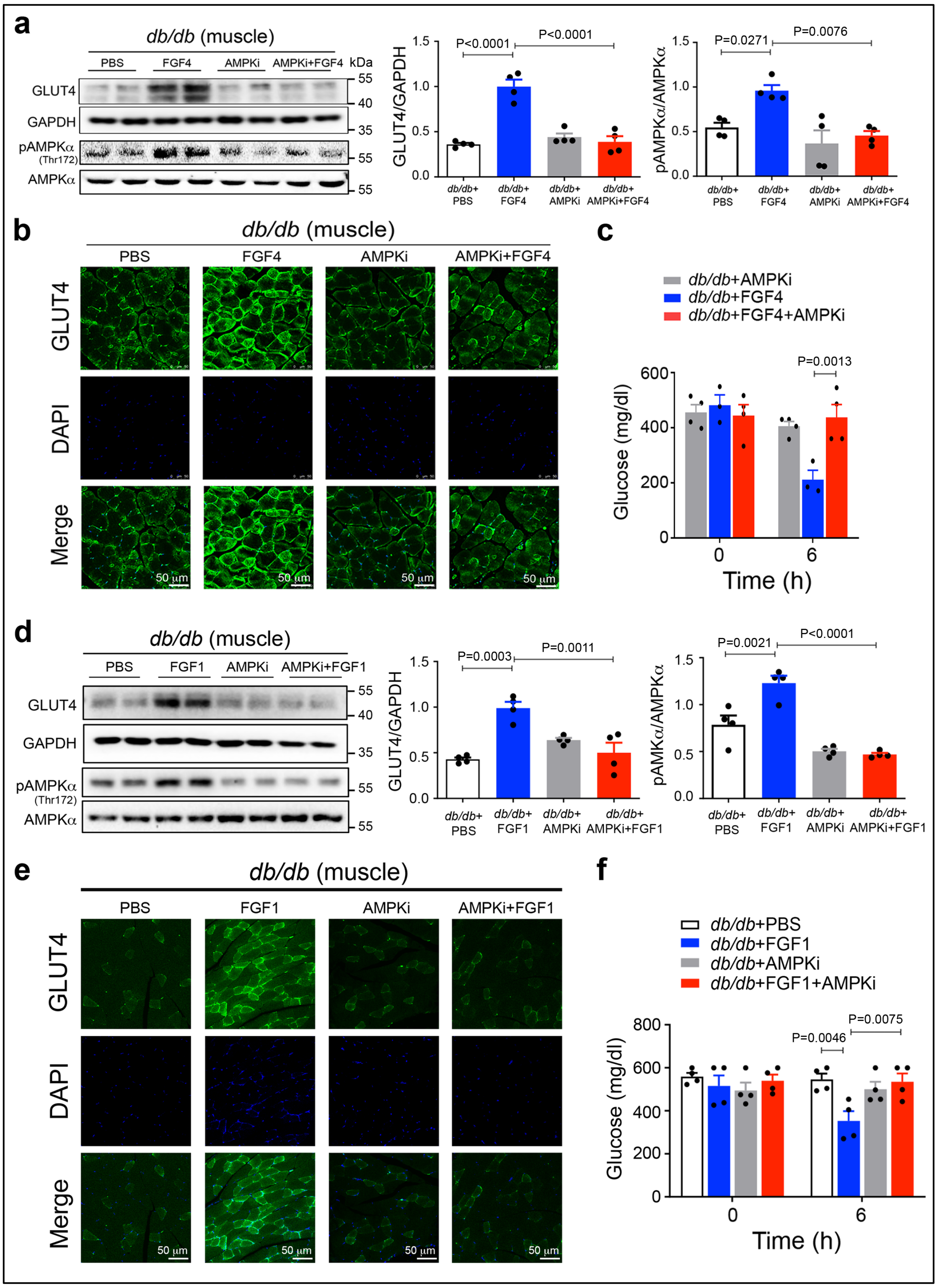

Supplementary Fig. 4. Both rFGF4 and rFGF1 lower blood glucose levels via activation of AMPKα signaling.

(a-f) *db/db* mice were pretreated with either Compound C (an AMPKα inhibitor) at 20 mg/kg body weight or a buffer control 1 hr before receiving an i.p. injection

of rFGF4 (**a-c**) or rFGF1 (**d-f**) (both at 1.0 mg/kg body weight). **(a, d)** AMPKα-mediated upregulation of GLUT4 expression in skeletal muscle 6 hrs after rFGF4 (a) or rFGF1 (d) injection as measured by western blot analysis (left hand panel) and quantitated using ImageJ software (right panels) (n=4). **(b, e)** AMPKα-mediated upregulation of GLUT4 expression and translocation in skeletal muscle 6 hrs after rFGF4 (b) or rFGF1 (e) injection as measured by immunofluorescence staining with an anti-GLUT4 antibody. Data are representative of 4 mice from each group. Scale bars, 50 μm. **(c, f)** Blood glucose levels of *db/db* mice 6 hrs after rFGF4 (c) (*db/db*+AMPKi (n=4), *db/db*+FGF4 (n=3), *db/db*+FGF4+AMPKi (n=4)) or rFGF1 (n=4) (f) injection. Data are presented as mean +/- SEM. Statistical comparisons in (a, d) are one-way ANOVA tests with Tukey's multiple comparisons tests. Statistical comparisons in (c, f) are two-way ANOVA tests with Tukey's multiple comparisons tests. Source data are provided as a Source Data file.

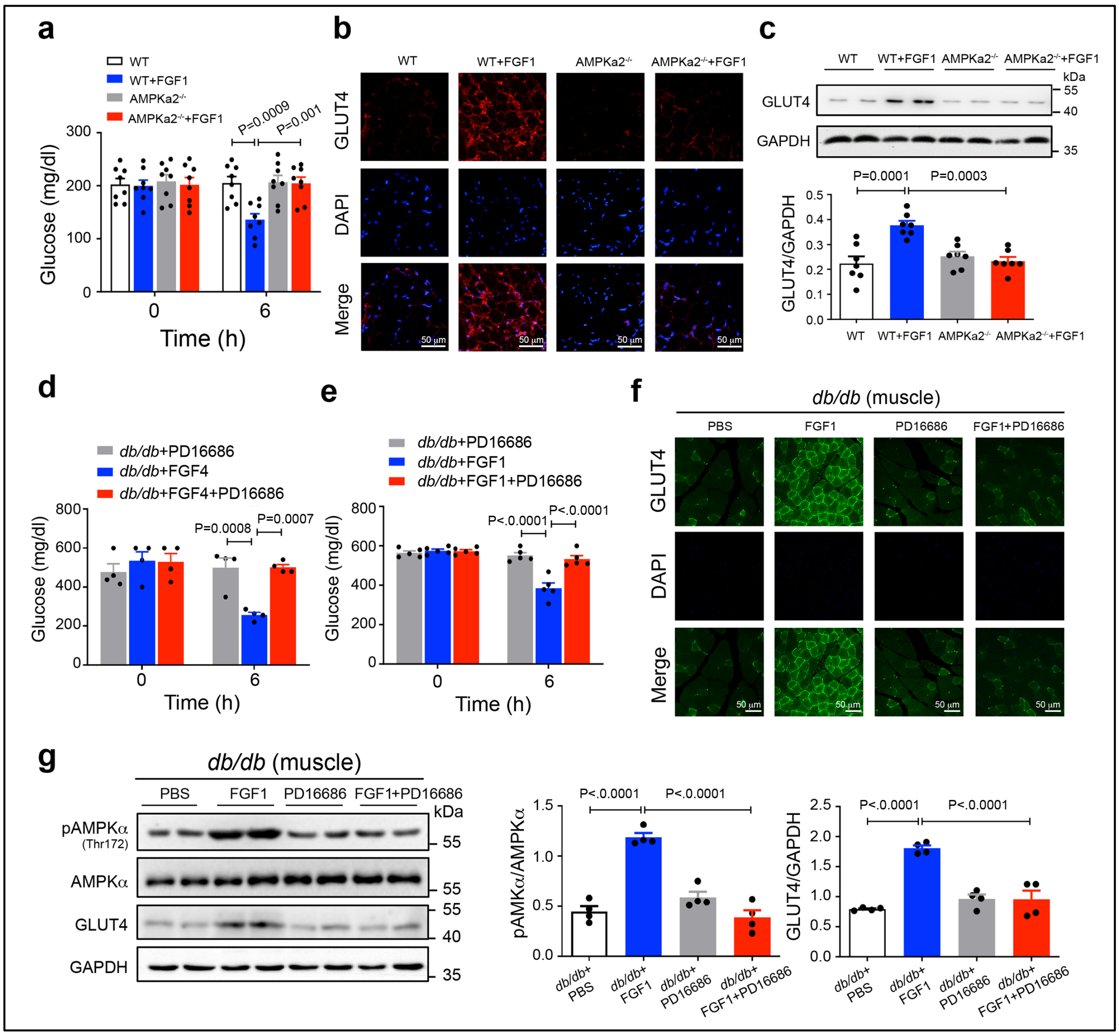

Supplementary Fig. 5. A single bolus of rFGF1 lowers blood glucose via FGFR-dependent activation of AMPK signaling.

(a) Blood glucose levels before and 6 hrs after i.p. injection of rFGF1 (1.0 mg/kg body weight) into wild type (WT) and AMPKα2 knockout mice (AMPKα2$^{-/-}$) fed 12 weeks long with a high-fat diet (n=8). **(b)** Expression and translocation of GLUT4 in LLM of AMPKα2$^{-/-}$ mice 6 hrs after i.p. injection of rFGF1 (1.0 mg/kg body weight) shown by immunofluorescence staining using an anti-GLUT4 antibody. Data are representative of 4 mice from each group. Scale bars, 50 μm. **(c)** GLUT4 expression in LLM of AMPKα2 knockout mice 6 hrs after i.p. injection of buffer (control) or rFGF1 (1.0 mg/kg body weight) as measured by

western blotting (left panel) and quantitated using ImageJ software (n=7). **(d)** Blood glucose levels of *db/db* mice 6 hrs after a single injection of rFGF4 (1.0 mg/kg body weight). Mice were pretreated with the FGFR1 inhibitor (PD166866) (20 mg/kg body weight) or a buffer control 1 hr before receiving an i.p. injection of rFGF4 (n=4). **(e-g)** *db/db* mice were pretreated with PD166866 at 20 mg/kg body weight or a buffer control 1 hr before receiving an i.p. injection of rFGF1 (1.0 mg/kg body weight). **(e)** Blood glucose levels of *db/db* mice 6 hrs after rFGF1 injection (n=5). **(f)** FGFR1-mediated upregulation of GLUT4 expression and translocation in skeletal muscle 6 hrs after rFGF1 injection as measured by immunofluorescence staining with an anti-GLUT4 antibody. Data are representative of 4 mice from each group. Scale bars, 50 μm. **(g)** Phosphorylation of AMPKα and expression of GLUT4 in skeletal muscle 6 hrs after rFGF1 injection as measured by western blot analysis and quantitated using ImageJ software (n=4). Data are presented as mean +/- SEM. Statistical comparisons in (a, d and e) are two-way ANOVA tests with Tukey's multiple comparisons tests. Statistical comparisons in (c, g) are one-way ANOVA tests with Tukey's multiple comparisons tests. Source data are provided as a Source Data file.

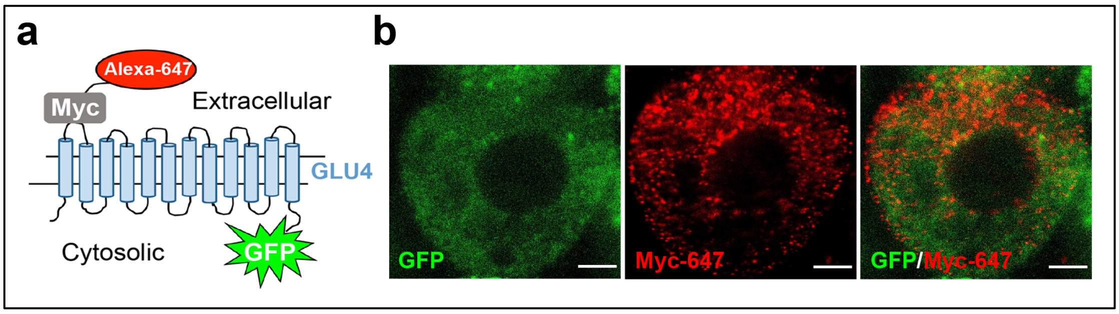

Supplementary Fig. 6. Analysis of GLUT4 localization in 3T3-L1 adipocytes.

(a) Schematics of Myc-GLUT4-GFP fusion protein used to study GLUT4 localization. The fusion protein carries a Myc epitope tag, inserted in its first exofacial loop and a GFP, added in frame to its the carboxy terminal tail. **(b)** Detection of cell surface expressed GLUT4 through anti-Myc fluorescence immunolabelling and total GLUT4 through GFP fluorescence in non-permeabilized 3T3-L1 adipocytes transfected with Myc-GLUT4-GFP expression plasmid. Scale bars, 5 µm.

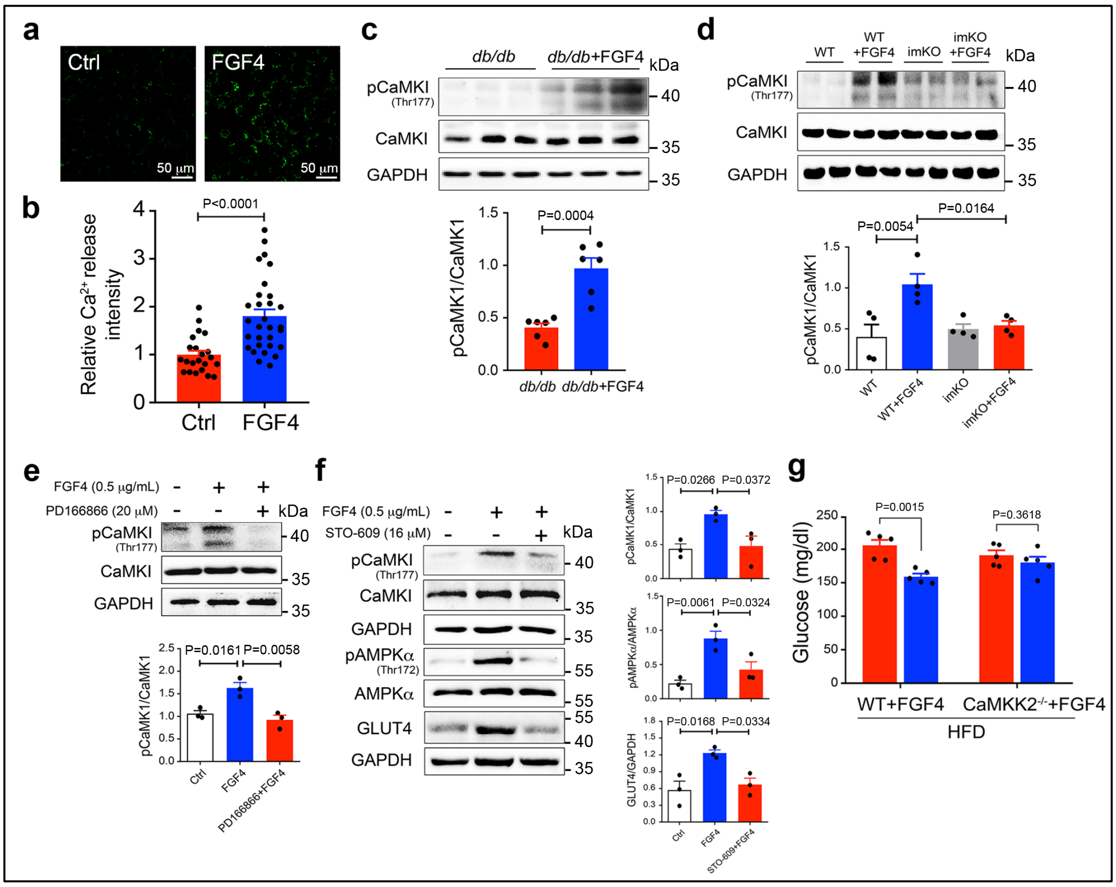

Supplementary Fig. 7. rFGF4 enhances GLUT4 expression via activation of the CaMKK2/AMPKα signaling pathway downstream of FGFR1.

(a,b) Calcium release in mouse embryonic fibroblasts treated with rFGF4 (0.5 μg/mL) (n=30) or a buffer control (n=22) for 10 mins detected by immunofluorescence with Fluo4-AM as a calcium indicator. Scale bars, 50 μm. **(c,d)** Phosphorylation levels of CaMKI in lower limb muscle (LLM) of *db/db* mice (**c**) (n=6) or inducible skeletal muscle FGFR1 knockout (imKO) mice (**d**) (n=4) 6 hrs after i.p. injection of rFGF4 (1.0 mg/kg body weight) or buffer as determined by western blotting (upper panels) and quantitated using ImageJ software (lower panels). **(e)** CaMKI phosphorylation levels in differentiated rat myoblasts cells (L6) - pretreated with PD166866 and then exposed to rFGF4 - as determined by western blotting (upper panel) and semi-quantitated using

ImageJ software (lower panel). Data from three independent measurements are presented as mean +/- SEM. **(f)** Semi-quantitative western blot analyses of CaMKI and AMPKα phosphorylation levels and GLUT4 expression in L6 cells pretreated with STO-609 and then exposed to rFGF4. Data from three independent measurements are presented as mean +/- SEM. **(g)** Blood glucose levels 6 hrs after i.p. injection of rFGF4 (1.0 mg/kg body weight) into WT and CaMKK2 knockout mice (CaMKK2$^{-/-}$) (n=5). The mice in panel d and g were fed with high-fat diet (HFD) for 12 weeks. Data are presented as mean +/- SEM. Statistical comparisons in (b, c and g) are unpaired two-tailed tests. Statistical comparisons in (d-f) are one-way ANOVA tests with Tukey's multiple comparisons tests. Source data are provided as a Source Data file.

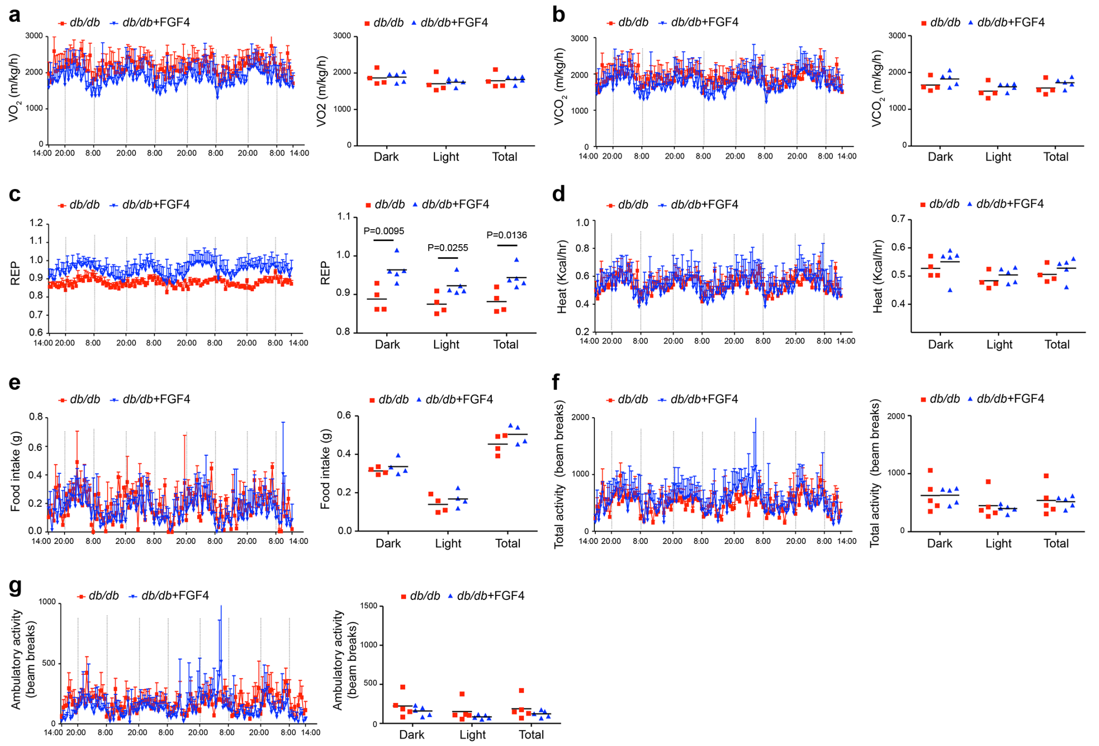

Supplementary Fig. 8. Long-term treatment with rFGF4 does not affect energy consumption or physical activity of *db/db* mice.

(a-g) Oxygen consumption (VO_2) (**a**) (*db/db* (n=4), *db/db*+FGF4 (n=5)), carbon dioxide production (VCO_2) (**b**) (*db/db* (n=4), *db/db*+FGF4 (n=5)), respiratory exchange ratio (RER) (**c**) (*db/db* (n=4), *db/db*+FGF4 (n=5)), heat production (**d**) (*db/db* (n=4), *db/db*+FGF4 (n=5)), food intake (**e**) (n=4), and total **(f)** (n=5) and ambulatory activity (**g**) (n=5) in chronically rFGF4-treated (administered on alternate days for 37 days; 1.0 mg/kg body weight) *db/db* mice (blue) and buffer treated controls (red). Data are presented as mean +/- SEM. Statistical comparison in (c) is unpaired two-tailed test. Source data are provided as a Source Data file.

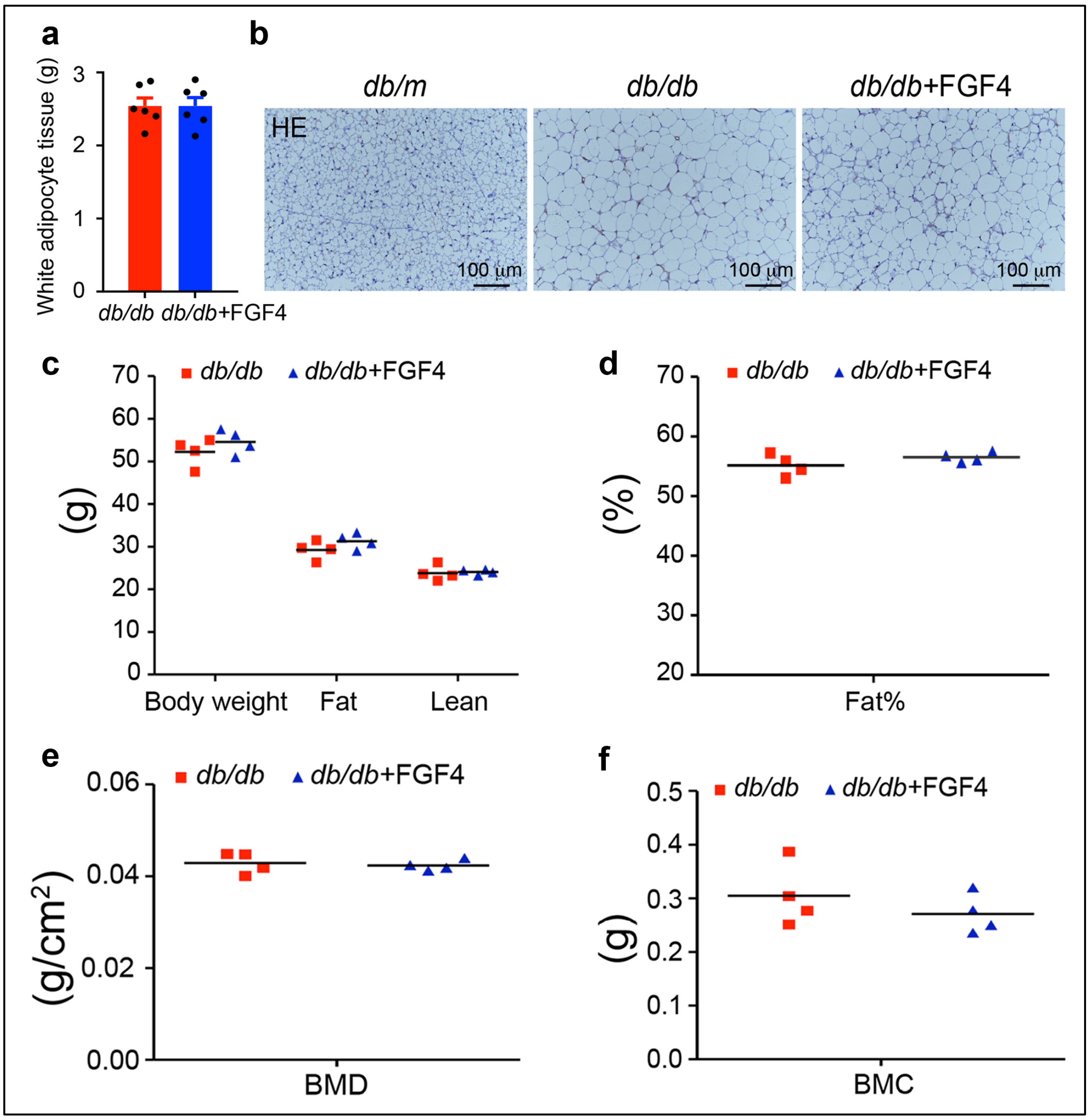

Supplementary Fig. 9. Long-term treatment with rFGF4 does not impact Epi-WAT morphology, body composition or bone density in *db/db* mice.

(a-f) Various parameters were measured in *db/db* mice after administration on alternate days for 37 days with either rFGF4 (1.0 mg/kg body weight) or a buffer control. **(a,b)** Weight **(a)** and representative haematoxylin and eosin (H&E) staining of Epi-WAT **(b)** (n=6). **(c,d)** Total body mass, fat and lean weight **(c)** and body fat percentage **(d)** (n=4). **(e,f)** Bone mineral density (BMD) **(e)** and bone mineral content (BMC) **(f)** (n=4). Data are presented as mean +/- SEM. Source data are provided as a Source Data file.

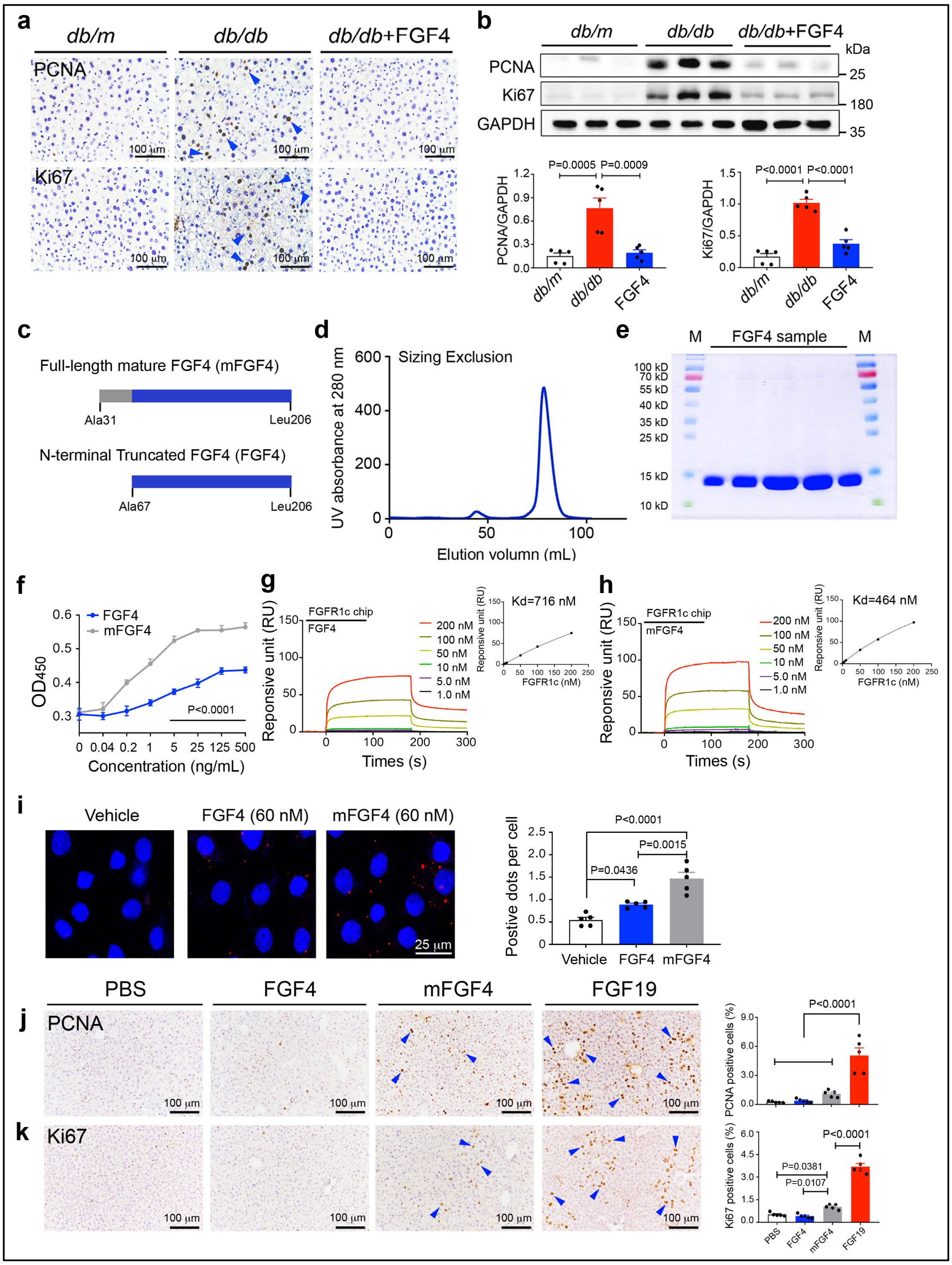

Supplementary Fig. 10. Long-term treatment with rFGF4 does not induce hepatocyte proliferation in experimental mice.

(a,b) Representative immunohistochemical staining **(a)** and expression of PCNA and Ki67 by western blotting of liver extracts from *db/db* mice using specific antisera **(b)** after administration on alternate days for 37 days with either rFGF4

(1.0 mg/kg body weight) or a buffer control (*db/db*) (n=5). Semi-quantitative analyses of the western blot data presented in **(b)** are shown below, with GAPDH used as a loading control. Scale bar, 100 μm. **(c)** Linear representations of mature human FGF4 (i.e., Ala31-Leu206) (mFGF4, top) and the N-terminally truncated FGF4 variant (Ala67-Leu206) (rFGF4, below). **(d)** Purification by gel filtration of rFGF4. **(e)** Analysis by SDS-PAGE of fractions contained in the major peak shown in (d). **(f)** Dose-response curves showing proliferation of HepG2 cells treated with rFGF4 or mFGF4 (0-500 ng/ml). Data from three independent measurements are presented as mean +/- SEM. **(g, h)** Representative SPR sensorgrams of binding interactions of rFGF4 and mFGF4 with the extracellular ligand-binding domain of FGFR1c (left hand side) and the saturation binding curves used to derive equilibrium dissociation constants (K_d) (right hand side). **(i)** Representative fluorescence microscopy images from proximity ligation assay comparing FGFR1 dimerization abilities of rFGF4 an mFGF4 at 60 nM (left hand side) and quantitative representation of the results by counting the number of positive dots per nucleus (n=5). Scale bar, 25 μm. **(j,k)** Representative immunohistochemical staining of PCNA **(j)** and Ki67 **(k)** in the livers of wild type mice after daily administration for 15 days with rFGF4, mFGF4, rFGF19 (all at 1.0 mg/kg body weight) or a buffer control (n=5). Data were quantified using ImageJ software. Scale bar, 100 μm. Blue arrowheads in panels a, f and g represent cells stained with anti-PCNA or Ki67 antiserum. Data are presented as mean +/- SEM. Statistical comparisons in (b, i, j and k) are one-way ANOVA tests with Tukey's multiple comparisons tests. Statistical comparison in (f) is two-way ANOVA test with Šídák's multiple comparisons test. Source data are provided as a Source Data file.

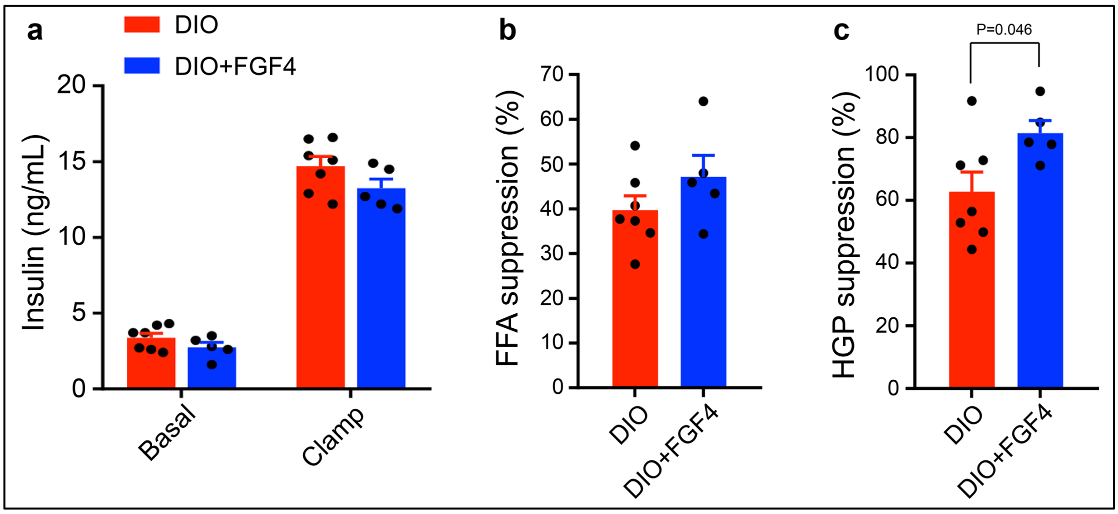

Supplementary Fig. 11. Long-term treatment with rFGF4 does not alter insulin level or free fatty acid (FFA) suppression but inhibits hepatic glucose production in DIO mice.

(a-c) Insulin level **(a)** and free fatty acid (FFA) **(b)** and hepatic glucose production (HGP) **(c)** suppressions during a hyperinsulinemic-euglycemic clamp test in DIO mice after administration on alternate days for 14 days with rFGF4 (1.0 mg/kg body weight) (n=5) or a buffer control (DIO) (n=7). Data are presented as mean +/- SEM. Statistical comparison in (c) is unpaired two-tailed test. Source data are provided as a Source Data file.

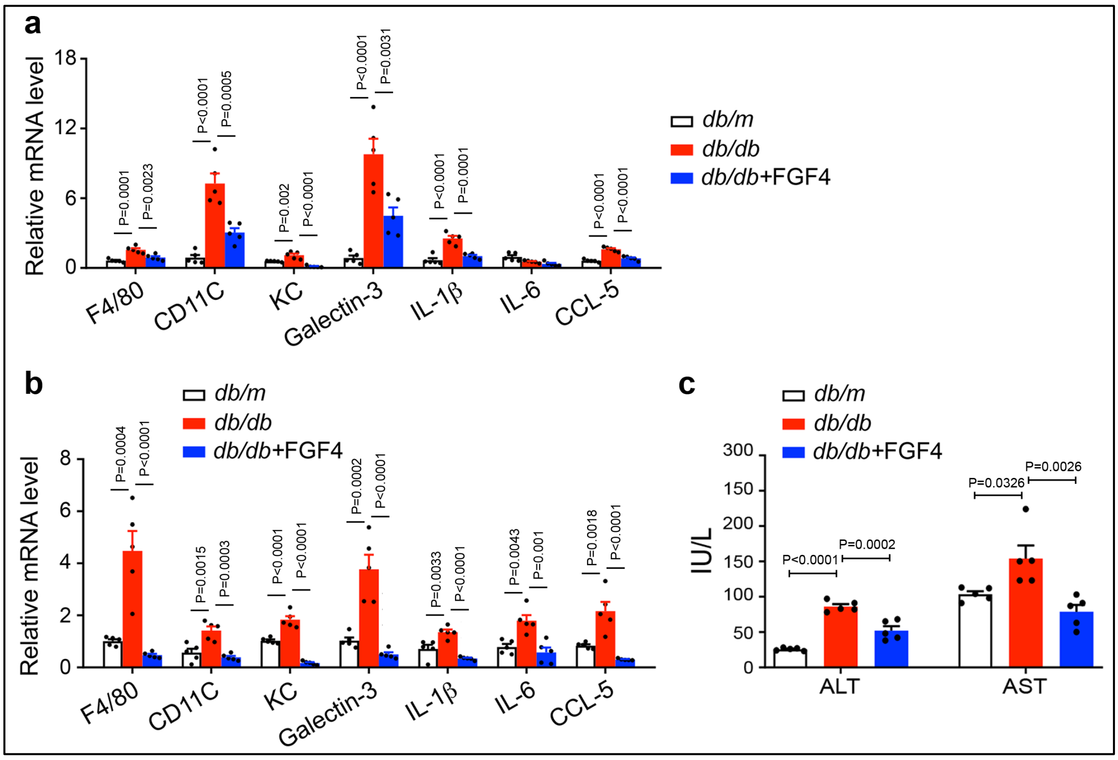

Supplementary Fig. 12. Long-term administration of rFGF4 suppresses inflammation in liver and skeletal muscle in *db/db* mice.

(a,b) mRNA expression levels of *F4/80, CD11C, KC, Galectin-3, IL-1β, IL-6* and *CCl5* in skeletal muscle **(a)** and livers **(b)** of *db/db* mice after 37 days of chronic treatment with rFGF4 (1.0 mg/kg body weight) or a buffer control (*db/db*) as determined by real-time PCR. Littermate *db/m* mice served as controls (n=5). **(c)** Serum levels of ALT and AST in *db/db* mice after 37 days of chronic treatment with rFGF4 (1.0 mg/kg body weight) or a buffer control (*db/db*); Littermate *db/m* mice served as controls (n=5). Data are presented as mean +/- SEM. Statistical comparisons in (a-c) are one-way ANOVA tests with Tukey's multiple comparisons tests. Source data are provided as a Source Data file.

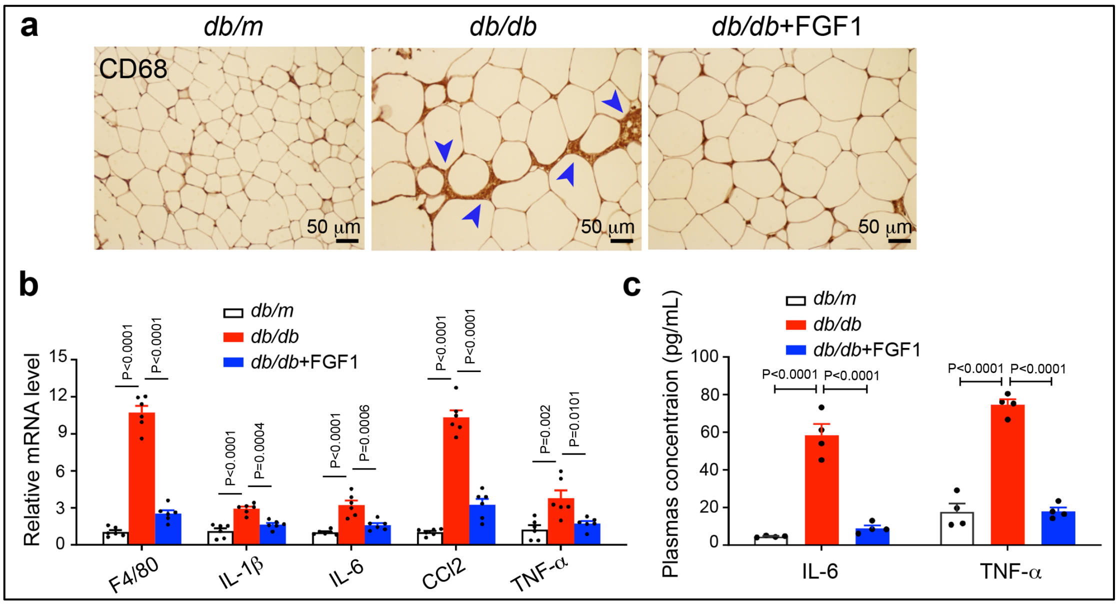

Supplementary Fig. 13. Long-term rFGF1 treatment improves local and systemic inflammation in *db/db* mice.

(a-c) Analysis of Epi-WAT tissue and sera from *db/db* mice treated over the course of 37 days with rFGF1 (1.0 mg/kg body weight). **(a)** Immunohistochemical staining with an anti-CD68 antiserum. Blue arrowheads represent cells stained with anti-CD68 antiserum. Data are representative of 4 mice from each group. Scale bar, 50 μm. **(b)** Real-time PCR analysis of expression of *F4/80, IL-1β, IL-6, CCl2* and *TNF-α* mRNAs. Littermate *db/m* mice served as controls (n=6). **(c)** Serum concentrations of TNF-α and IL-6 determined by ELISA (n=4). Data are presented as mean +/- SEM. Statistical comparisons in (b, c) are one-way ANOVA tests with Tukey's multiple comparisons tests. Source data are provided as a Source Data file.

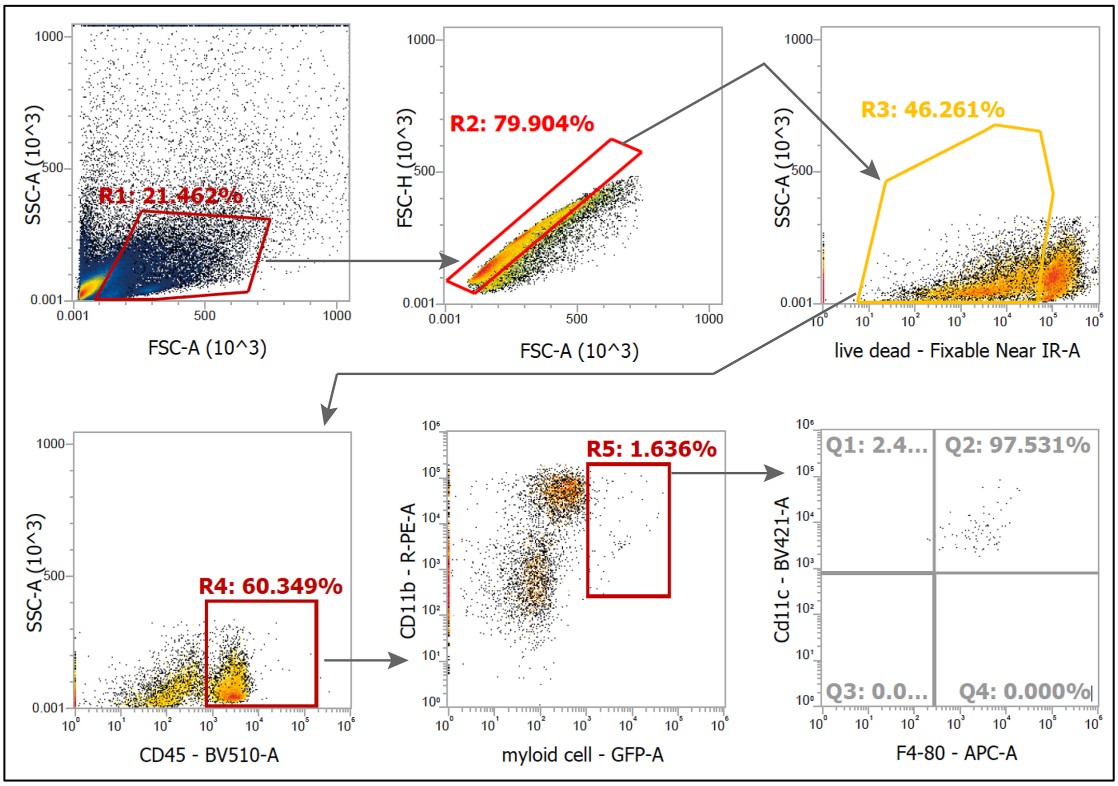

Supplementary Fig.14. Flow cytometry gating strategy.

Flow cytometry gating strategy used for detections of CD45$^+$CD11b$^+$GFP$^+$ monocytes and CD45$^+$CD11b$^+$GFP$^+$F4/80$^+$CD11c$^+$ macrophages in mouse stromal vascular cells from epididymal white adipose tissue.

Supplementary Experimental Procedures

Expression and purification of human recombinant FGF4

Recombinant full length human FGF1 (rFGF1) (Met1-Asp155), full length mature form human FGF4 (mFGF4) (Ala31-Leu206), full length mature human FGF7 (rFGF7) (Cys32-Thr194), FGF8 (rFGF8) (Gln23-Arg215), FGF9 (rFGF9) (Met1-Ser208), FGF19 (rFGF19) (Leu25-Lys216) and FGF21 (rFGF21) (His29-Ser209) were expressed and purified according to published protocols [1-7]. The extracellular D2–D3 region of human FGFR1c (Asp142-Arg365) was expressed in *E. coli* BL21 (DE3), refolded *in vitro* from inclusion bodies and further purified as previously described [8]. Expressed and purification of rFGF4 protein was achieved as follows. The cDNA fragment encoding residues Ala67-Leu206 of full length human FGF4 was amplified by PCR and subcloned into the the *NcoI* and *XhoI* restriction sites of vector pET-15b via. Competent *E. coli* BL-21 (DE3) cells transformed with the rFGF4 expression construct were cultured in 1 L LB medium containing 2% glucose and 30 mg/mL kanamycin in an incubator shaker at 37 °C and 200 rpm. At an optical density of 0.8-1.0 at λ_{600}, recombinant protein expression was induced by addition of isopropyl-L-thio-β-D-galactopyranoside (IPTG) to 1 mM and further growth at 37 °C for 4 hrs. Cells were harvested and lysed in 25 mM Na/K phosphate buffer (pH 7.5) containing 300 mM NaCl using an Emulsiflex-C3 (Avestin, Inc., Ottawa, Ontario, Canada) high-volume homogenizer. rFGF4 was found primarily in the soluble fraction. The lysate was clarified by centrifugation at 15,000 x g for 30 mins at 4 °C, followed by purification using a heparin affinity column (Heparin Hitrap, GE Healthcare, Piscataway, NJ, column volume (CV) = 5 mL) equilibrated in buffer A (150mM NaCl, 25mM HEPES, pH 7.5). Bound

rFGF4 was eluted using a linear gradient of NaCl to 2M NaCl in 25mM HEPES (pH 7.5). Fractions containing the protein of interest as determined by analysis via 12% SDS-PAGE were pooled, concentrated and applied to a gel filtration column (Superdex™-75 GE Healthcare, Piscataway, NJ) run in buffer C (1M NaCl, 25mM Tris-HCl, PH 8.0). The purity of the recombinant protein was estimated to be 98%.

Surface Plasmon Resonance Spectroscopy

Real-time biomolecular interactions of mFGF4 and rFGF4 with FGFR1c ectodomain were analyzed using a BIA-core T200 system (GE Healthcare, Piscataway, NJ) in HBS-EP buffer (10 mM HEPES-NaOH, pH 7.4, 150 mM NaCl, 3 mM EDTA, 0.005% Surfactant P20). Ligand-binding region of FGFR1c was immobilized onto flow channels of a CM5 sensor chip using an amine coupling kit (GE Healthcare, Piscataway, NJ) as previously described[9]; Briefly, purified FGFR1c samples were passed over the activated chip in Na acetate buffer (pH 5.5) and immobilized to 200-480 response units (RU). Chip surfaces were blocked using 1M ethanolamine-HCl (pH 8.5). The control flow channel was prepared exactly the same but without the protein. Increasing concentrations of mFGF and rFGF4 were diluted with HBS-EP buffer and injected over the FGFR1c chip for 180 s at 50 µl/min; HBS-EP buffer was then flowed for 180 s to monitor dissociation at 50 µl/min. Sensor chips surfaces were regenerated by injecting 1.5 M NaCl in 10 mM HEPES-NaOH (pH 7.5) or 2.0 M NaCl in 10 mM sodium acetate (pH 4.5). For each injection, non-specific responses from the control

flow channel were subtracted from responses recorded for the mFGF4 or rFGF4 flow channel. Data were processed using BIA-Evaluation software (V 3.0, GE Healthcare, Piscataway, NJ) and equilibrium dissociation constants (K_d) were calculated from fitted saturation binding curves.

Functional evaluation of rFGF1, rFGF4 and rFGF21 in diabetic mice

Mice were randomized based on body weight and blood glucose levels. All drugs and controls were delivered by i.p. injection into mice unless specified otherwise. For time-course studies, blood samples were taken at various time-points from conscious, fed or fasted *db/db,* DIO or *ob/ob* mice after a single injection of either rFGF4, rFGF1, rFGF21 (all at 1.0 mg/kg body weight), or buffer alone. Blood samples were taken from tail veins and glucose measured using a FreeStyle complete blood glucose monitor (Abbott Diabetes Care Inc., Alameda, CA). Food intake was measured 24 hrs after a single injection.

To evaluate the effect of rFGF4 on blood glucose in normal C57BL/6J or *db/db* mice, blood samples were taken after a single injection of rFGF4 (1.0 or 3.0 mg/kg body weight) as described above. *Db/m* or C57BL/6J mice were treated with PBS as a control. To evaluate the effect of phloretin, compound C or PD166866 on rFGF1- or rFGF4-mediated glucose lowering, phloretin (200 mg/Kg body weight), compound C (20 mg/Kg body weight), PD166866 (20 mg/Kg body weight) or buffer was given to *db/db* mice 1 hr prior to a single injection of rFGF1 or rFGF4 (all at 1.0 mg/kg body weight). To evaluate the glucose-lowering effect of rFGF1 or rFGF4 in AMPKα2 knockout, CaMKK2 knockout or conditional skeletal muscle FGFR1 knockout animals, blood samples

were taken at various time points after a single injection of rFGF1 or rFGF4 (all at 1.0 mg/kg body weight) as described above.

For chronic efficacy evaluation, *db/db* mice or DIO mice were injected with rFGF1 or rFGF4 (all at 1.0 mg/kg body weight) on alternate days for either 37 days (*db/db*) or 15 days (DIO); injection with buffer (PBS) alone served as controls. Blood samples were taken by tail snip and glucose levels measured as described above. Following the final dose, oral glucose tolerance tests (OGTTs) were conducted after fasting overnight (12 h); *db/db* or DIO mice were orally challenged with a dextrose solution (1.5 g/kg body weight). Glucose levels were determined as described above. Area under the curve (AUC) for OGTTs was calculated by applying the trapezoid rule for the glucose tolerance curve using GraphPad Prism 7 software (GraphPad Software, San Diego, California). Serum alanine aminotransferase (ALT) and aspartate aminotransferase (AST) levels were measured using an automatic biochemical analyzer (Hitachi Auto Analyzer 7020, Hitachi Co. Ltd., Tokyo, Japan) according to the manufacturer's instructions. TNF-α and IL-6 plasma levels were measured using a MILLIPLEX MAP mouse metabolic hormone magnetic bead panel-metabolism multiplex assay (Merck Millipore, Darmstadt, Germany). Bone mineral density (BMD), bone mineral content (BMC) and body composition were measured using dual-energy x-ray absorptiometry (PIXImus2, GE lunar, Madison, WI).

Hyperinsulinemic-euglycemic clamp tests

db/db or DIO mice received either rFGF4 (1.0 mg/kg body weight) or buffer alone (as a control) on alternate days over a period of 2 weeks, and subjected to hyperinsulinemic-euglycemic clamp test as previously described[10] with minimal

modifications. Briefly, one catheter (Silastic 508-001, Dow Corning) was implanted in the right jugular vein of anesthetized mice and then tunneled subcutaneously to the back of the neck and exteriorized. Mice were allowed to recover for 4 days prior to clamp experiments. Animals losing <5% of their pre-surgery weight were included in the following test.

For DIO mice, following a 6 hour fast, blood was sampled at t=-90 min from the tail vein for determination of basal insulin and FFA levels. The clamp test was then started by infusion with D-[3-^{3}H] glucose (PerkinElmer Life Sciences, MA, USA) at a constant rate of 5 μCi/h for 90 min. After equilibration, a blood sample was taken for basal glucose turnover determination. Then glucose (50% D-glucose, variable infusion rate) and tracer (5 μCi/h) plus insulin (Humulin, Eli Lily and Company; 8 mU/kg/min) were infused into the jugular vein starting from t=0 min. Small blood samples were drawn from the tail vein at 10 min intervals and achievement of steady-state conditions (120 mg/dl ± 5 mg/dl) was confirmed at the end of the clamp by maintaining glucose infusion and plasma glucose concentration for a minimum of 30 min. Blood samples at t=0 (basal), 110 and 120 (end of experiment) min were taken to determine glucose-specific activity, FFA and insulin concentration. Tracer-determined rates were quantified using the Steele equation for steady-state conditions. At steady state, the rate of glucose disappearance, or total GDR, is equal to the sum of the rate of endogenous glucose productions (HGP) plus the exogenous (cold) GIR. The IS-GDR is equal to the total GDR minus the basal glucose turnover rate.

For *db/db* mice, blood was also sampled after a 6 hour fast to determine basal insulin and FFA levels. A primed bolus containing human insulin (500 mU/kg, Humulin R, Eli Lily, Indianapolis, IN) was injected and the clamp was

initiated by infusing only glucose (50% D-glucose, variable infusion rate) and insulin (Humulin, Eli Lily and Company. 20 mU/kg/min) at t=0 min. Small blood samples were drawn from the tail vein at 10 min intervals and achievement of steady-state conditions (120 mg/dl ± 5 mg/dl) was confirmed at the end of the clamp by maintaining glucose infusion and plasma glucose concentration for a minimum of 30 min. GIR was measured during the final 30 minutes of each step when a near steady state had been attained. Blood samples collected at t=110 and t=120 (end of experiment) min were used to determine steady state FFA and insulin concentration.

Pharmacokinetic evaluation

The *in vivo* half-life of rFGF1, rFGF4 or mFGF4 was determined following a single intraperitoneal (i.p.) injection (all at 1 mg/kg body weight) into adult male Sprague Dawley (SD) rats (220–250 g). Blood aliquots (200 uL) were drawn from the tail vein at various times over the course of 7 h. Protein levels were measured using a human FGF1 or FGF4 immunoassay ELISA kit (Cloud-Clone Corp, Wuhan, Hubei). Pharmacokinetic parameters of rFGF1, rFGF4 or mFGF4 were calculated using the Drug and Statistics Software (DAS, V 2.0; Mathematical Pharmacology Professional Committee of China). The elimination half-live (t1/2) was calculated using the formula t1/2 = 0.693/Ke, where Ke is the elimination rate constant.

RNA extraction, cDNA synthesis and quantitative RT-PCR

Total RNA was extracted from mouse tissues with TRIzol reagent (Thermo Fisher Scientific, Waltham, MA) and purified using an RNeasy Mini Kit (Qiagen,

Valencia, CA). A Two-step M-MLV Platinum SYBR Green qPCR Super Mix-UDG kit (Thermo Fisher Scientific, Waltham, MA) was used for reverse transcription and quantitative PCR. GAPDH or UBC was used as an endogenous control to normalize for differences in the amount of total RNA added to each reaction. Primers (shown in **Supplementary Table 4**) were synthesized by Sangon Biotech Co., Ltd (Shanghai， China).

Pathological, histopathological, immunohistochemical and immunofluorescent evaluation of mouse tissues

Epi-WAT, liver or skeletal muscle tissues were excised from WT, FGFR1$^{flox/flox}$, *db/m*, *db/db*, conditional skeletal muscle FGFR1 knockout, or AMPKα2 knockout mice following acute or chronic administration of PBS, rFGF1, rFGF21, rFGF4, mFGF4 or rFGF19 (all at 1.0 mg/kg body weight) and weighed. Epi-WAT, liver and skeletal muscle were fixed in 4% paraformaldehyde overnight or in 75% acetone/25% ethanol for 5 mins and embedded in either paraffin or using Tissue-Tek OCT compound (Sakura, Tokyo, Japan). After deparaffinization and rehydration, paraffin sections (5 μm) were stained with haematoxylin and eosin (H&E) reagent using standard procedures. Skeletal muscle glycogen content was visualized by periodic acid–Schiff (PAS) staining of paraffin sections.

For immunohistochemistry, Epi-WAT paraffin sections were incubated overnight at 4 °C with primary antibody (rabbit polyclonal to CD68 (1:200), from Abcam Cambridge, MA). Liver paraffin sections were incubated overnight at 4 °C with primary antibody (mouse monoclonal to PCNA (1:500), from Santa Cruz biotechology, Dallas, Texas; or rabbit polyclonal to Ki67 (1:500), from Abcam Cambridge, MA). After washing, Epi-WAT or liver sections were

incubated with horseradish peroxidase-conjugated secondary antibody against rabbit (1:200) or mouse (1:200) (all from Santa Cruz Biotechnology, Dallas, Texas, USA), developed with a DAB (3,3-diaminobenzidine) developing system (Beyotime Biotechnology, Shanghai, China), counterstained with hematoxylin and observed by light microscopy (Nikon, Tokyo, Japan).

For immunofluorescence, lower limb muscles were excised from C57BL/6J, *db/db*, FGFR1$^{flox/flox}$, AMPKα2 knockout and conditional skeletal muscle FGFR1 knockout mice 6 hrs after a single administration of PBS (as a control), rFGF1, rFGF21 or rFGF4 (all at 1.0 mg/kg body weight). Paraffin or frozen sections of muscle were incubated overnight at 4 °C with primary antibody (mouse to GLUT4 (1:200), from Abmart Biotechnology, Shanghai, China or rabbit polyclonal to GLUT4 (1:100), from Novus Biologicals, Centennial, CO), followed by incubation with a goat anti-mouse lgG AF 594 secondary antibody (1:200 dilution) (Abmart Biotechnology, Shanghai, China) or a donkey anti-rabbit IgG H&L (Alexa Fluor® 488) secondary antibody (1:1000 dilution) (Abcam, Cambridge, UK) at room temperature for 1 h followed by a 10 min incubation with 40,6-diamidino-2-phenylindole (DAPI) (SouthernBiotech, Birmingham, AL). In some experiments, DAPI (Beyotime Biotechnology, Shanghai, China) was added to the secondary antibody (goat anti-mouse lgG AF 594) at 0.5 μg/ml. For immunofluorescent double-staining, Epi-WAT paraffin sections were incubated overnight at 4 °C with both a rat monoclonal anti-F4/80 antibody (FITC) (1:200) (Abcam, Cambridge, UK) and an Armenian hamster monoclonal anti-CD11C antibody (1:200) (Abcam, Cambridge, UK). A goat anti-Armenian hamster IgG H&L (Alexa Fluor® 647) (Abcam, Cambridge, UK) secondary antibody (1:1000 dilution) was used at room temperature for 1 h.

Immunofluorescence images were obtained with a fluorescence microscope (Zeiss, Oberkochen, Germany) or a confocal microscope (Leica, Mannheim, Germany).

Experiments using L6 muscle cells

All cell-based assays were done using L6 rat skeletal myoblasts (Serial number: GNR 4, National Collection of Authenticated Cell Cultures of China, Shanghai, China). Cells were cultured in DMEM supplemented with 100 IU/mL penicillin, 100 mg/mL streptomycin sulfate and 10% FBS (all from Thermo Fisher Scientific, Waltham, MA) in a 5% CO_2 incubator at 37 °C. Differentiation and myotube formation were induced by growing cells to 80-90% confluence and switching the medium to 2% FBS for 7-8 days.

For glucose uptake assay, cells were treated with rFGF4 (0.1 or 0.5 μg/mL), insulin (100 nM) or buffer control for 1 hr and subjected to a glucose uptake assay according to the manufacturer's instructions (Promega, Madison, Wisconsin). To measure activation of intracellular signaling pathways, cells were rinsed with PBS buffer and treated with rFGF4 (0.1 or 0.5 μg/mL), insulin (50 nM) or rFGF4 plus insulin for 5, 10, 15 or 20 mins at 37 °C. For studies of an insulin resistant state, cells were pretreated with insulin (200 nM), insulin plus rFGF4 (0.5 μg/mL -pre) or buffer alone for 2 hrs. Cells were then stimulated with insulin (50 nM), rFGF4 (0.5 μg/mL) or insulin plus rFGF4 for 20 mins. To establish the effects of inhibitors on rFGF4-induced intracellular pathways, cells were pretreated with the FGFR1 antagonist PD166866 (20 μM) or the CaMKK2 selective antagonist STO-609 (16 μM) for 30 mins and exposed to rFGF4 (0.5 μg/ml) for an additional 20 mins.

For knockdown of AMPKα2 expression by siRNA, cells were seeded in 6-well plates and allowed to grow to 70% confluence for 24 h. Transient transfections were performed using Lipofectamine RNAiMAX (Thermo Fisher Scientific, Waltham, MA) according to the manufacturer's protocol. After transfection with either siRNA (control or AMPKα2 siRNA) (all from Santa Cruz Biotechnology, Dallas, Texas, USA) for 24 h, cells were grown to 80-90% confluence and the culture medium switched to 2% FBS for 7-8 days to induce differentiation and myotube formation.

Proximity ligation assay

L6 rat skeletal myoblasts ectopically expressing human FGFR1 were grown on glass slides at 1.5×10^5/slide and placed in 12-well plates. Cultured cells were starved for 8 hrs in serum-free DMEM medium and treated with rFGF4 or mFGF4 (both at 60 nM) for 15 min. The cells were fixed with 4% paraformaldehyde for 30 mins, washed with PBS and blocked with PBS containing with 5% BSA for 1 hr at 37 °C. Cells were then incubated with a mixture of two different anti-FGFR1 primary antibody (Cat#ab824, mouse monoclonal antibody, from Abcam, Cambridge, UK and Cat#9740, rabbit monoclonal antibody, from Cell Signaling Technology, Danvers, MA) at 4 °C overnight. The proximity ligation assay (PLA) reaction was performed according to the manufacturer's instructions provided (Cat#DUO92101, from Sigma, Burlington, Vermont). Cells were incubated with diluted PLUS and MINUS PLA probes at 37 °C for 1 hr. The ligation of the two probes was achieved by incubation with ligase for 30 min. Cells were then

incubated with amplification solution for 100 min, followed by incubation with Duolink PLA Mounting Medium containing DAPI for 15 mins. The immunofluorescence images were acquired using a confocal microscope (Leica, Mannheim, Germany).

GLUT4 translocation analysis by total internal reflection fluorescence (TIRF) microscopy

TIRF microscopy was used to study GLUT4 translocation in the context of 3T3-L1 mouse embryonic fibroblasts (Resource number: ATCC® CRL-3242™, ATCC, Manassas, Virginia) transfected with a Myc-GLUT4-GFP expression plasmid (gift from Dr. Xiaowei Chen from Peking University). By choosing a certain angle of excitation laser (59.6°), TIRF imaging can strictly ensure the fluorescent intensity emanated only from the narrow zone near cellular surface [11].

3T3-L1 cells were cultured in high-glucose DMEM supplemented with 50 IU/mL penicillin, 50 mg/mL streptomycin sulfate, 10% FBS and 1% 100×Glutamate in a 5% CO_2 incubator at 37 °C and allowed to reach confluence. Two days later, adipocyte differentiation was induced by switching the medium into differentiation medium containing 10% FBS, 2 mg/mL insulin, 0.25 mM dexamethasone and 0.5 mM 3-isobutyl-1-methylxanthine. The differentiated adipocytes were transfected with Myc-GLUT4-GFP expression plasmid using electroporation and cultured in DMEM supplemented with 50 IU/mL penicillin, 50 mg/mL streptomycin sulfate, 3% FBS, and 1% 100×Glutamate for 2 days to allow expression of Myc-GLUT4-GFP. The transfected adipocytes were treated with rFGF4 (0.5 μg/mL), insulin (100 nM) or a buffer control for 20 mins. The

cells were fixed with 4% paraformaldehyde, washed with PBS and then blocked with PBS containing with 5% goat serum for at least 1 h. Cells were then incubated with primary antibodies (mouse monoclonal antibody to Myc (1:1000), from Cell Signaling Technology, Danvers, MA) at 4 °C overnight, followed by incubation with goat anti-Mouse IgG H&L (Alexa Fluor® 647) secondary antibody (Thermo Fisher Scientific, Waltham, MA) at room temperature for 1 hr. The immunofluorescence and TIRF images showing profoundly punctate expression of GLUT4 were acquired using a confocal microscope (Zeiss, Oberkochen, Germany).

Bone marrow-derived macrophage (BMM) experiments

Isolation/culture of primary BMMs from C57BL/6J mice was performed as previously described[12]. Cells were seeded in 6-well plates at 2×10^6 cells/ml in modified BMM medium (containing macrophage colony-stimulating factor (M-CSF) (PeproTech, Rocky Hill, NJ) / low glucose DMEM, 100 IU/mL penicillin, 100 mg/mL streptomycin sulfate and 10% FBS (all from Thermo Fisher Scientific, Waltham, MA) and maintained in a 5% CO_2 incubator at 37 °C. Cells were differentiated by replacing the BMM medium every 1-2 d. After seven days of differentiation, the cells were used in the following studies.

For western blot analysis, cultured cells were starved for 24 hrs in low glucose and serum-free DMEM medium, pretreated with rFGF4 (1, 10, or 100 ng/ml) for 1 hr, and incubated in high glucose (HG) (36 mM) for 2 hrs. Cells were lysed and the levels of various downstream signals were detected by western blotting (for details, see western blotting methods).

For NF-κB subunit P65 nuclear import assays, cultured cells were treated as

described above. After stimulation, cells were fixed with 4% paraformaldehyde and permeabilized with 100 % methanol at -20 °C for 5 min. Cells were washed twice with PBS containing 1% BSA and incubated with primary antibodies (rabbit monoclonal antibody to NF-κB p65 (1:200), from Cell Signaling Technology, Danvers, MA) at 4 °C overnight, followed by incubation with donkey anti-rabbit IgG H&L (Alexa Fluor® 647) secondary antibody (Abcam, Cambridge, UK) at room temperature for 1 hr. Cells were incubated with DAPI for 10 min and immunofluorescence images acquired using a confocal microscope (Leica, Mannheim, Germany).

For measurements on IL-6 levels, cultured cells were starved, pretreated with serum free medium containing rFGF4 and incubated in high glucose (HG) as described above. IL-6 levels in the culture medium were determined by ELISA according to the manufacturer's instructions (Cloud-Clone Corp., Katy, TX).

Supplementary References

1 Beenken, A., Eliseenkova, A. V., Ibrahimi, O. A., Olsen, S. K. & Mohammadi, M. Plasticity in interactions of fibroblast growth factor 1 (FGF1) N terminus with FGF receptors underlies promiscuity of FGF1. *J Biol Chem* **287**, 3067-3078, doi:10.1074/jbc.M111.275891 (2012).

2 Bellosta, P. *et al.* Identification of receptor and heparin binding sites in fibroblast growth factor 4 by structure-based mutagenesis. *Mol Cell Biol* **21**, 5946-5957 (2001).

3 Sher, I., Yeh, B. K., Mohammadi, M., Adir, N. & Ron, D. Structure-based mutational analyses in FGF7 identify new residues involved in specific interaction with FGFR2IIIb. *FEBS Lett* **552**, 150-154 (2003).

4 Olsen, S. K. *et al.* Structural basis by which alternative splicing modulates the organizer activity of FGF8 in the brain. *Gene Dev* **20**, 185-198, doi:10.1101/gad.1365406 (2006).

5 Kalinina, J. *et al.* Homodimerization controls the fibroblast growth factor 9 subfamily's receptor binding and heparan sulfate-dependent diffusion in the extracellular matrix. *Mol Cell Biol* **29**, 4663-4678, doi:10.1128/MCB.01780-08 (2009).

6 Goetz, R. *et al.* Molecular insights into the klotho-dependent, endocrine mode of action of fibroblast growth factor 19 subfamily members. *Mol Cell Biol* **27**, 3417-3428, doi:10.1128/Mcb.02249-06 (2007).
7 Kurosu, H. *et al.* Tissue-specific expression of betaKlotho and fibroblast growth factor (FGF) receptor isoforms determines metabolic activity of FGF19 and FGF21. *J Biol Chem* **282**, 26687-26695, doi:10.1074/jbc.M704165200 (2007).
8 Plotnikov, A. N., Hubbard, S. R., Schlessinger, J. & Mohammadi, M. Crystal structures of two FGF-FGFR complexes reveal the determinants of ligand-receptor specificity. *Cell* **101**, 413-424, doi:Doi 10.1016/S0092-8674(00)80851-X (2000).
9 Niu, J. *et al.* Curtailing FGF19's mitogenicity by suppressing its receptor dimerization ability. *Proc Natl Acad Sci U S A* **117**, 29025-29034, doi:10.1073/pnas.2010984117 (2020).
10 Li, P. *et al.* Hematopoietic-Derived Galectin-3 Causes Cellular and Systemic Insulin Resistance. *Cell* **167**, 973-984 e912, doi:10.1016/j.cell.2016.10.025 (2016).
11 Lizunov, V. A., Matsumoto, H., Zimmerberg, J., Cushman, S. W. & Frolov, V. A. Insulin stimulates the halting, tethering, and fusion of mobile GLUT4 vesicles in rat adipose cells. *J Cell Biol* **169**, 481-489, doi:10.1083/jcb.200412069 (2005).
12 Weischenfeldt, J. & Porse, B. Bone Marrow-Derived Macrophages (BMM): Isolation and Applications. *CSH Protoc* **2008**, pdb prot5080, doi:10.1101/pdb.prot5080 (2008).

Supplementary Table S1. Pharmacokinetics of rFGF1, rFGF4 and mFGF4 in SD rats injected intraperitoneally with 1.0 mg/kg of body weight

Parameters	rFGF4	mFGF4	rFGF1
AUC(0-t)/μg/L*h	1134.323±14.646	609.500±8.617	448.010±51.327
AUC (0-∞)/μg/L*h	1956.879±70.492	1037.722±35.562	590.940±82.368
R_AUC (t/ ∞)/%	58.380±2.988	59.020±2.185	76.820±1.818
t1/2z/h	4.445±0.353	4.300±0.330	2.515±0.111
Tmax/h	3.800±0.374	3.600±0.245	1.500±0.387
Vz/F/L/kg	3.260±0.171	5.952±0.296	6.494±0.676
CLz/F/L/h/kg	0.514±0.021	0.968±0.032	1.821±0.235
C_{max}/μg/L	203.483±4.668	112.869±3.103	115.990±11.735

Data are presented as mean ± SEM (n=5).

Supplementary Table 2. Organ weights of *db/db* mice chronically treated with rFGF4.

	db/m	***db/db***	***db/db*+rFGF4**	
Heart weight /tibia length	63.1+/-3.3	75.0+/-4.0	76.6+/-8.1	mg/cm
Liver weight /tibia length	580.2+/-26.2	1990.3+/-73.4$^{####}$	1711.3+/-70.5*	mg/cm
Left kidney /tibia length	92.4+/-3.9	120.0+/-9.1	127+/-12.2	mg/cm
Right kidney /tibia length	96.7+/- 6.7	113.9+/-7.8	130.7+/-6.5	mg/cm
Spleen weigh /tibia length	39.4+/- 3.9	78.1+/-22.9	58.0+/-17.5	mg/cm

Ad libitum-fed *db/db* mice were injected with rFGF4 (1.0 mg/kg body weight) (n=3) on alternate days for 37 days with PBS buffer (*db/db*) (n=3); *db/m* mice served as controls (n=6). Data are presented as mean +/- SEM. $^{####}$p<0.0001, *db/db* vs *db/m*; *p<0.05 (P=0.0152, *db/db*+rFGF4 vs *db/db*). Statistical comparisons are one-way ANOVA tests with Tukey's multiple comparisons tests.

Supplementary Table 3. Experimental reagents and antibodies.

REAGENT or RESOURCE	SOURCE	IDENTIFIER	
**Antibodies**			**Dilution**
Glut4 (1F8) Mouse mAb antibody	Cell Signaling Technology	Cat#2213<br>RRID: AB_823508<br>(For *Fig. 2g, Fig. 3f, Fig.4, Fig.6a&e, Fig.S1f, Fig.S4a&d, Fig.S5g and Fig. S7f*)	WB (1:1000)
Glucose Transporter GLUT4 Antibody	Novus Biologicals	Cat# NBP1-49533<br>RRID: AB_10011583	IF (1:100)
GLUT4 antibody	Proteintech	Cat#66846-1-Ig<br>RRID: AB_2882186<br>(For *Fig. 3d and Fig. S5c*)	WB (1:1000)
GLUT4 antibody	Abmart Biotechnology	Cat#MB1001<br>RRID: N/A	IF (1:200)
Phospho-FGF Receptor (Tyr653/654) Antibody	Cell Signaling Technology	Cat#3471S<br>RRID: AB_331072	WB (1:1000)
FGF Receptor 1 (D8E4) XP Rabbit mAb antibody	Cell Signaling Technology	Cat#9740S<br>RRID: AB_11178519	PLA (1:100)<br>WB (1:1000)
FGFR1 antibody [M5G10]	Abcam	Cat#ab824<br>RRID: AB_306385	PLA (1:100)
Phospho-AMPKα (Thr172) (40H9) Rabbit mAb	Cell Signaling Technology	Cat#2535<br>RRID: AB_331250	WB (1:1000)
AMPKα (D5A2) Rabbit mAb	Cell Signaling Technology	Cat#5831<br>RRID: AB_10622186	WB (1:1000)
AMPKalpha2 Antibody	Cell Signaling Technology	Cat#2757<br>RRID: AB_560858	WB (1:1000)
Rabbit Anti-CaMKI, phospho (Thr177) Polyclonal Antibody	Abcam	Cat# ab62215<br>RRID: AB_940775	WB (1:800)
Rabbit Anti-CaMKI Monoclonal Antibody	Abcam	Cat# ab68234<br>RRID: AB_1140889	WB (1:1000)
Anti-phospho-IRS1	Merck Millipore	Cat#09-432	WB (1:800)

(Tyr608) mouse/ (Tyr612) human antibody		RRID: AB_1163457	
IRS-1 Antibody	Cell Signaling Technology	Cat#2382 RRID: AB_330333	WB (1:1000)
Phospho-Akt (Ser473) antibody	Cell Signaling Technology	Cat#4060 RRID: AB_2315049	WB (1:1000)
Akt (pan) (C67E7) Rabbit mAb antibody	Cell Signaling Technology	Cat#4691 RRID: AB_915783	WB (1:1000)
Phospho-Glycogen Synthase (Ser641) Antibody	Cell Signaling Technology	Cat#3891 RRID: AB_2116390	WB (1:1000)
Glycogen Synthase Antibody	Cell Signaling Technology	Cat#3893 RRID: AB_2279563	WB (1:1000)
GAPDH (14C10) Rabbit mAb	Cell Signaling Technology	Cat#2118 RRID: AB_561053	WB (1:1000)
Myc-Tag (9B11) Mouse mAb	Cell Signaling Technology	Cat#2276 RRID: AB_331783	IF (1:1000)
Ki67 antibody	Abcam	Cat# ab15580 RRID: AB_443209	WB (1:1000) IHC (1:500)
PCNA (F-2) antibody	Santa Cruz Biotechology	Cat# sc-25280 RRID: AB_628109	WB (1:1000) IHC (1:500)
CD68 antibody	Abcam	Cat#ab125212 RRID: AB_10975465	WB (1:1000) IHC (1:200)
F4/80 antibody [BM8] (FITC)	Abcam	Cat#ab60343 RRID: AB_941505	IF (1:200)
CD11c antibody [N418]	Abcam	Cat#ab33483 RRID: AB_726084	IF (1:200)
MCP-1 (ECE.2) antibody	Santa Cruz Biotechology	Cat#sc-52701 RRID: AB_628867	WB (1:1000)
Phospho-SAPK/JNK (Thr183/Tyr185) (81E11) Rabbit mAb antibody	Cell Signaling Technology	Cat#4668S RRID: AB_823588	WB (1:1000)
SAPK/JNK (56G8) Rabbit mAb antibody	Cell Signaling Technology	Cat#9258S RRID: AB_10694056	WB (1:1000)
p-IkappaB-alpha (B-9) antibody	Santa Cruz Biotechnology	Cat#sc-8404 RRID: AB_627773	WB (1:1000)
Phospho-IKK / (Ser176/180) (16A6) Rabbit	Cell Signaling Technology	Cat# 2697 RRID: AB_2079382	WB (1:1000)

mAb antibody			
NF-κB p65 (D14E12) XP Rabbit mAb	Cell Signaling Technology	Cat#8242<br>RRID: AB_10860244	IF (1:200)
goat anti-rabbit IgG-HRP antibody	Santa Cruz Biotechnology	Cat#sc-2004<br>RRID: AB_631746	WB (1:3000)<br>IHC (1:200)
goat anti-mouse IgG-HRP antibody	Santa Cruz Biotechnology	Cat#sc-2005<br>RRID: AB_631736	WB (1:3000)<br>IHC (1:200)
Donkey Anti-Rabbit IgG H&L (Alexa Fluor® 488) antibody	Abcam	Cat#ab150073<br>RRID: AB_2636877	IF (1:1000)
Goat Anti-Armenian hamster IgG H&L (Alexa Fluor® 647)	Abcam	Cat#ab173004<br>RRID: AB_2732023	IF (1:1000)
Donkey Anti-Rabbit IgG H&L (Alexa Fluor® 647)	Abcam	Cat#ab150075<br>RRID: AB_2752244	IF (1:1000)
Goat anti-Mouse IgG H&L (Alexa Fluor® 647)	Thermo Fisher Scientific	Cat# A32728<br>RRID: AB_2633277	IF (1:1000)
Goat Anti-Mouse lgG AF 594	Abmart Biotechnology	Cat#M21013<br>RRID: N/A	IF (1:200)
Brilliant Violet 510 anti-mouse CD45 antibody	Biolegend	Cat#103138<br>RRID: AB_2563061	FCM (1:1000)
PE anti-mouse/human CD11b antibody	Biolegend	Cat#101208<br>RRID: AB_312791	FCM (1:1000)
F4/80 Monoclonal Antibody (BM8), APC	Thermo Fisher Scientific	Cat#17-4801-82<br>RRID: AB_2735035	FCM (1:1000)
Brilliant Violet 421 anti-mouse CD11c antibody	Biolegend	Cat#117329<br>RRID: AB_10897814	FCM (1:1000)
**Chemicals, Peptides, and Recombinant proteins**			
BL-21 (DE3)	Transgen Biotech	Cat#CD601	
Kanamycin	Transgen Biotech	Cat#GG201-01	
Isopropyl-L-thio-β	Sigma-Aldrich	Cat#I6758	

-D-galactopyranoside		
Hitrap Heparin HP, 5ml	GE healthcare	Cat#17-5112-01
SuperdexTM-75	GE healthcare	Cat#28-9893-33
HBS-EP buffer	GE healthcare	Cat#BR-1006-69
Series S sensor chip CM5	GE healthcare	Cat#BR-1005-30
DMEM High Glucose	Thermo Fisher Scientific	Cat#11995065
DMEM Low Glucose	Thermo Fisher Scientific	Cat#11885084
Fetal Bovine Serum (FBS)	Thermo Fisher Scientific	Cat#10099-141
Goat Serum	Beyotime Biotechnology	Cat#C0265
Penicillin-Streptomycin, Liquid	Thermo Fisher Scientific	Cat#15140122
HEPES (1 M)	Thermo Fisher Scientific	Cat#15630106
Insulin (dry powder)	Sigma-Aldrich	Cat#91077C
Insulin (liquid) Humulin R	Eli Lilly	Lot#C583597C
Recombinant Murine M-CSF	PeproTech	Cat#315-02
Collagenase from Clostridium histolyticum (Type II-S)	Sigma-Aldrich	Cat#C1764
RIPA Lysis and Extraction Buffer	Thermo Fisher Scientific	Cat#89900
T-PER Tissue Protein Extraction Reagent	Thermo Fisher Scientific	Cat#78510
Protease Inhibitor Cocktail	Thermo Fisher Scientific	Cat#78430
Phosphatase Inhibitor Cocktail	Thermo Fisher Scientific	Cat#78420
Periodic Acid-Schiff stain Kit	LEAGENE	Cat#DG0005
Minute™ Plasma Membrane Protein	Invent Biotechnologies,	Cat#SM-005

Isolation Kit	Inc	
Hematoxylin and Eosin Staining Kit	Beyotime Biotechnology	Cat#C0105
Bovine serum albumin	Sigma-Aldrich	Cat#B2064
Enhanced chemiluminescence reagents	Bio-Rad	Cat#1705040
Phosphate Buffered Saline (PBS)	Thermo Fisher Scientific	Cat#10010023
Phosphate Buffered Saline (PBS)	Beyotime Biotechnology	Cat#C0221A
DAB Horseradish Peroxidase Color Development Kit	Beyotime Biotechnology	Cat#P0203
DAPI Fluoromount-G®	SouthernBiotech	Cat#0100-20
DAPI	Beyotime Biotechnology	Cat#C1002
Antifade Mounting Medium	Beyotime Biotechnology	Cat#P0126
Fluo-4, AM	Molecular Probes	Cat#F14201
Phloretin	Sigma-Aldrich	Cat#P7912
Dorsomorphin (Compound C) 2HCl	Selleck	Cat#S7306
PD166866	Selleck	Cat#S8493
STO-609	Selleck	Cat#S8274
TRIzol	Thermo Fisher Scientific	Cat#15596026
RNeasy Mini Kit	Qiagen	Cat#74106
Platinum™ SYBR™ Green qPCR SuperMix-UDG w/ROX	Thermo Fisher Scientific	Cat#11744100
FcR Blocking Reagent, mouse	Miltenyi Biotec. Inc	Cat#130-092-575
EasySep™ Mouse Monocyte Isolation Kit	STEMCELL Tech	Cat#19761
eBioscience™ 10X	Thermo Fisher	00-4300-54

RBC Lysis Buffer (Multi-species)	Scientific	
**Critical Commercial Assays**		
ELISA Kit for Fibroblast Growth Factor 1, Acidic (FGF1)	Cloud-Clone Corp.	Cat#SEA032Hu
ELISA Kit for Fibroblast Growth Factor 4 (FGF4)	Cloud-Clone Corp.	Cat#SEA034Hu
ELISA Kit for Interleukin 6 (IL6)	Cloud-Clone Corp.	Cat#SEA079Mu
Glycogen Assay Kit	Abcam	Cat#ab65620
Rat/Mouse Insulin ELISA Kit	Merck Millipore	Cat#EZRMI-13K
Glucose Uptake-Glo™ Assay	Promega	Cat#J1342
MILLIPLEX MAP Mouse Metabolic Hormone Magnetic Bead Panel - Metabolism Multiplex Assay	Merck Millipore	Cat#MMHMAG-44K-07
Duolink® In Situ Red Starter Kit Mouse/Rabbit	Sigma-Aldrich	Cat#DUO92101
BCA Protein Assay Kit	Beyotime Biotechnology	Cat#P0009
**Experimental Models: Cell Lines**		
L6 rat skeletal myoblasts	National Collection of Authenticated Cell Cultures of China	Serial number: GNR 4
3T3-L1 MBX	ATCC	Resource number: ATCC® CRL-3242™
HepG2 cell	Dr. Shengcai Lin (the School of Life Sciences, Xiamen University)	N/A
**Experiment Models: Organisms/Strains**		

C57BL/6J mice	Model Animal Research Center of Nanjing University, China	N/A
db/db mice (C57BLKS/J-lepr^{db} /lepr^{db})	Model Animal Research Center of Nanjing University, China	N/A
db/m mice (C57BLKS/J)	Model Animal Research Center of Nanjing University, China	N/A
ob/ob mice (C57BL/6J-lep^{ob}/lep^{ob})	Model Animal Research Center of Nanjing University, China	N/A
EGFP mice (STOCK-Tg (CAG-EGFP)/Nju)	Model Animal Research Center of Nanjing University, China	N/A
Sprague Dawley rats	Model Animal Research Center of Nanjing University, China	N/A
FGFR1$^{flox/flox}$ mice (B6.129S4-Fgfr1$^{tm5.1Sor}$/J)	The Jackson Laboratory	Stock No. 007671
AMPKα2 knockout mice (C57BL/6)	Dr. Louise D. McCullough (Department of Neurology, McGovern Medical School at The University of Texas Health Science Center at Houston (UTHealth))	N/A
CaMKK2 knockout mice	Dr. Shengcai Lin (the School of Life Sciences, Xiamen University)	N/A
**Oligonucleotides**		

Real-time PCR primers used in this study	See **Supplementary Table 4**	N/A
**Software and Algorithms**		
GraphPad Prism 7	GraphPad	https://www.graphpad.com/
Image J, V 1.42q	National Institutes of Health (NIH)	https://imagej.nih.gov/ij/
BIA-Evaluation software, V 3.0	GE Healthcare	
Inveon Research Workplace 4.2	Siemens	
Flow Jo, V 10	FlowJo, LLC	https://www.flowjo.com/
DAS, V 2.0	Mathematical Pharmacology Professional Committee of China	
**Other**		
Tissue Tek OCT Compound	SAKURA	Cat#4583
Standard chow diet	Lab Diet	Cat#5053
High-fat diet (60 % fat)	Research Diets, Inc	Cat#D12492
Cell culture dish (100mm)	Corning	Cat#430167
Six-well plate	Corning	Cat#3516
96-well plate	Corning	Cat#3599
Cell cryotube(2.0ml)	Corning	Cat#430659
Falcon® 70 μm Cell Strainer	Corning	Cat#352350

Western Blot (WB), Immunohistochemistry (IHC), Immunofluorescence (IF), Proximity ligation assay (PLA), Flow Cytometry (FCM).

Supplementary Table 4. Sequences of the primers used for real-time PCR in this study.

Gene Name	Forward (5'-3')	Reverse (5'-3')
GLUT4	GTGACTGGAACACTGGTCCTA	CCAGCCACGTTGCATTGTAG
Klb	TGTTCTGCTGCGAGCTGTTAC	CCGGACTCACGTACTGTTTTT
Fgfr1c	GGTGCTTCATCTACGGAATGTCTCC	TCGGAGACTCCAGCCAGCATGG
Fgfr2b	TAAATAGCTCCAATGCAGAAGTGC	GGTGTCCGCTGTTGAGGACAGACG
Fgfr2c	GGAATGTAACTTTTGAGGATGCTGG	GGTGTCCGCTGTTGAGGACAGACG
Fgfr3b	CAATTTCATAGGCGTGGCTGAGAAGG	CTTGTCGATGCCAATAGCTTCTGC
Fgfr3c	ACACCACCGACAAGGAGCTAGAGG	CTTGTCGATGCCAATAGCTTCTGC
Fgfr4	CCTGAAGACAACAGACATCAATAGC	GTTGATGATGTTCTTGTGTCTTCCG
Pkm	GCCGCCTGGACATTGACTC	CCATGAGAGAAATTCAGCCGAG
Hk2	TGATCGCCTGCTTATTCACGG	AACCGCCTAGAAATCTCCAGA
Shda	GGAACACTCCAAAAACAGACCT	CCACCACTGGGTATTGAGTAGAA
Pdha1	GAAATGTGACCTTCATCGGCT	TGATCCGCCTTTAGCTCCATC
Pdk4	AGGGAGGTCGAGCTGTTCTC	GGAGTGTTCACTAAGCGGTCA
F4/80	CTTTGGCTATGGGCTTCCAGTC	GCAAGGAGGACAGAGTTTATCGTG
CD11c	ACACAGTGTGCTCCAGTATGA	GCCCAGGGATATGTTCACAGC
CCL2	TTAAAAACCTGGATCGGAACCAA	GCATTAGCTTCAGATTTACGGGT
CCL3	TGTACCATGACACTCTGCAAC	CAACGATGAATTGGCGTGGAA
CCL5	GCTGCTTTGCCTACCTCTCC	TCGAGTGACAAACACGACTGC
CXCL10	CCAAGTGCTGCCGTCATTTTC	TCCCTATGGCCCTCATTCTCA
TNFα	GGCATGGATCTCAAAGACAACC	AAATCGGCTGACGGTGTGG
Galectin-3	ATGAAGAACCTCCGGGAAAT	GCTTAGATCATGGCGTGGTT
KC	ACTGCACCCAAACCGAAGTC	TGGGGACACCTTTTAGCATCTT
IL-1β	AAATACCTGTGGCCTTGGGC	CTTGGGATCCACACTCTCCAG
IL-6	TAGTCCTTCCTACCCCAATTTCC	TTGGTCCTTAGCCACTCCTTC
GAPDH	AATGTGTCCGTCGTGGATCT	CATCGAAGGTGGAAGAGTGG
UBC	GCCCAGTGTTACCACCAAGAAG	GCTCTTTTTAGATACTGTGGTGAGGAA
